# Causal Impact of Type 2 Diabetes Mellitus on Cerebral Small Vessel Disease: A Mendelian Randomization Analysis

**DOI:** 10.1161/STROKEAHA.117.020536

**Published:** 2018-04-23

**Authors:** Junfeng Liu, Loes Rutten-Jacobs, Ming Liu, Hugh S. Markus, Matthew Traylor

**Affiliations:** 1 From the Stroke Clinical Research Unit, Department of Neurology, West China Hospital, Sichuan University, Chengdu, P. R. China (J.L., M.L.); 2 Department of Clinical Neurosciences, Stroke Research Group, University of Cambridge, United Kingdom (J.L., L.R.-J., H.S.M., M.T.); 3 Population Health Sciences, German Center for Neurodegenerative Diseases (DZNE), Bonn, Germany (L.R.-J.).

**Keywords:** cerebral small vessel disease, diabetes mellitus, type 2, insulin resistance, Mendelian randomization analysis, stroke, lacunar

## Abstract

**Background and Purpose—:**

The relationship between type 2 diabetes mellitus (T2D) and cerebral small vessel disease (CSVD) is unclear. We aimed to examine the causal effect of T2D, fasting glucose levels, and higher insulin resistance on CSVD using Mendelian randomization.

**Methods—:**

Five CSVD phenotypes were studied; 2 were clinical outcomes associated with CSVD (lacunar stroke: n=2191/27 297 and intracerebral hemorrhage [ICH]: n=2254/8195 [deep and lobar ICH]), whereas 3 were radiological markers of CSVD (white matter hyperintensities: n=8429; fractional anisotropy [FA]: n=8357; and mean diffusivity: n=8357). We applied 2 complementary analyses to evaluate the association of T2D with CSVD. First, we used summarized data from genome-wide association study to calculate the effects of T2D-related variants on CSVD with inverse-variance weighted and weighted median approaches. Second, we performed a genetic risk score approach to test the effects of T2D-associated variants on white matter hyperintensities, FA, and mean diffusivity using individual-level data in UK Biobank.

**Results—:**

T2D was associated with higher risk of lacunar stroke (odds ratio [OR], 1.15; 95% confidence interval [CI], 1.04–1.28; *P*=0.007) and lower mean FA (OR, 0.78; 95% CI, 0.66–0.92; *P*=0.004) but not white matter hyperintensities volume (OR, 1.01; 95% CI, 0.97–1.04; *P*=0.626), higher mean diffusivity (OR, 1.04; 95% CI, 0.89–1.23; *P*=0.612), ICH (OR, 1.07; 95% CI, 0.95–1.20; *P*=0.269), lobar ICH (OR, 1.07; 95% CI, 0.89–1.28; *P*=0.466), or deep ICH (OR, 1.16; 95% CI, 0.99–1.36; *P*=0.074). Weighted median and penalized median weighted analysis showed similar effect estimates of T2D on lacunar stroke and FA, but with wider CIs, meaning they were not significant. The genetic score on individual-level data was significantly associated with FA (OR, 0.63; 95% CI, 0.45–0.89; *P*=0.008) after adjusting for potential confounders.

**Conclusions—:**

Our Mendelian randomization study provides evidence to suggest that T2D may be causally associated with CSVD, in particular with lacunar stroke and FA.

## Abstract 2

Supplemental Digital Content is available in the text.

Cerebral small vessel disease (CSVD) is an age-related disease affecting the small blood vessels of the brain.^1,2^ It accounts for at least 25% of all strokes and is the most common cause of vascular dementia.^1,2^ Several neuroimaging features are associated with CSVD, including lacunar infarcts, white matter hyperintensities (WMH), enlarged perivascular spaces, microbleeds, and brain atrophy. Intracerebral hemorrhages (ICH), particularly those arising from deep perforating small vessels, are also believed to be caused by CSVD.^2^ In addition, diffusion tensor imaging measures, such as fractional anisotropy (FA) and mean diffusivity (MD), are thought to capture microstructural changes of the white matter related to CSVD.^3^

However, the pathogenesis of CSVD is still uncertain,^2^ and consequently few effective and mechanism-based treatments for CSVD are available, aside from management of vascular risk factors of CSVD.^2^ Understanding which vascular risk factors are truly causal and improved understanding of how these pathological processes relate to different neuroimaging features of CSVD has the potential to improve treatment and prevention of CSVD.

Type 2 diabetes mellitus (T2D) is an established risk factor for ischemic stroke and cognitive decline.^4,5^ Epidemiological studies have suggested that T2D is associated with lacunar stroke,^6,7^ but the relationships of T2D with WMH, ICH, and other radiological markers of CSVD have been inconsistent.^4,6–9^ Such studies are also limited by the study design, which can be confounded. Therefore, a definitive causal association between T2D and CSVD is yet to be established. In addition, few studies have specifically studied the relationship between higher insulin resistance and fasting glucose levels and risk of CSVD,^10–12^ leaving a gap in knowledge regarding whether either is the driving force behind increased risk of CSVD.

Mendelian randomization (MR), using genetic variants as instrumental variables, is a method that enables stronger claims to be made about the causality of risk factors in disease pathogenesis.^13^ It is based on the theory that genetic variants are randomly allocated at meiosis, similar to a randomized controlled trial.^14^ Therefore, genetic variants are independent of many other factors that bias observational studies, such as confounding and reverse causation. In the absence of pleiotropy, a significant association in an MR study between an exposure and outcome implies causality. In the present study, we aimed first to use MR to determine whether T2D is causally associated with clinical outcomes associated with CSVD; lacunar stroke and ICH; as well as intermediate radiological markers of CSVD; WMH, FA and MD. Second, we performed exploratory analyses investigating the relationship between higher insulin resistance and fasting glucose levels and risk of CSVD. All analyses are based on the aggregate effects of genetic variants rather than clinically diagnosed T2D.

## Methods

The data that support the findings of this study are available from the corresponding author on reasonable request.

### Study Design, Data Sources, and Ethical Approval

We performed a MR analysis, testing the causal relationship of T2D, fasting glucose, and fasting insulin with 5 manifestations of CSVD (magnetic resonance imaging–confirmed lacunar stroke, ICH [alone and stratified by the location of hemorrhage: deep ICH and lobar ICH], WMH, FA, and MD). Analyses of all CSVD phenotypes were based on subjects of European ancestry only.

For ICH, we used a data set composed of 2254 cases and 8195 controls from 3 studies. One thousand five hundred forty-five cases and 1481 controls were from the Intracerebral Hemorrhage Genetics Collaboration and downloaded from the Cerebrovascular Disease Knowledge Portal (http://cerebrovascularportal.org).^15^ Five hundred seventy-five cases and 5750 propensity score–matched controls (matched on age, sex, and ancestry-informative principal components) were from UK Biobank, based on algorithmically defined ICH in White British subjects.^16^ One hundred thirty four cases and 964 controls were from the Cambridge ICH Genetics Study. For full details, Methods section in the online-only Data Supplement.

The magnetic resonance imaging–confirmed lacunar stroke data were derived from 2191 lacunar stroke cases with magnetic resonance imaging confirmation from the SIGN-NINDS,^17^ WTCCC2,^18^ and DNA lacunar genome-wide association studies and 27 297 controls.^19^ All cases were obtained based on hospital admissions.

Radiological markers of CSVD were derived from UK Biobank. Procedures for brain imaging acquisition and initial quality check have been described previously and are available on the UK Biobank website (Brain Imaging Documentation V1.3, http://www.ukbiobank.ac.uk).^20^ For FA and MD, we analyzed the first principal component of mean FA or MD values across 48 standard space tracts in 8357 individuals. WMH data were based on an analysis of 8429 subjects from UK Biobank. Additional details are provided in the Methods section in the online-only Data Supplement.

Study characteristics for each of the data sets are provided in Table 1.

**Table 1.**
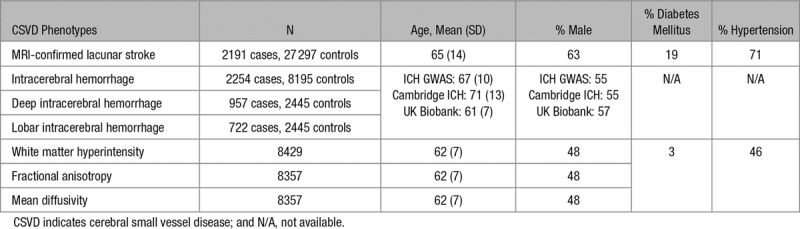
Cohort Characteristics

An IRB or regional review board has approved the use of human subjects in each of the study populations. All patients gave informed consent. UK Biobank received ethical approval from the research ethics committee (REC reference 11/NW/0382). The present analyses were conducted under UK Biobank application number 19463.

### Single-Nucleotide Polymorphism Selection

We selected single-nucleotide polymorphisms (SNPs) associated with T2D, fasting insulin, and fasting glucose, which reached genome-wide significance in the largest genome-wide association meta-analyses to date.^21–23^ Eighty-four SNPs were included for T2D, 36 for fasting glucose, and 18 for fasting insulin (Table I in the online-only Data Supplement). In instances where SNPs were not available in a data set because of poor imputation quality, we replaced them with proxy SNPs if available (*r*^2^>0.7). We included only 1 SNP from any associated locus. We verified that SNPs were uncorrelated by performing LD clumping (*r*^2^>0.1, 100 kb) using PLINK.^24^

### Statistical Analyses

We performed 2 complementary analyses to evaluate the impact of T2D-associated variants on CSVD phenotypes. For our primary analysis of the association of T2D with all CSVD phenotypes using 2-sample MR on summary statistics, we used a significance threshold of *P*<0.0071, equivalent to Bonferroni correction for 7 independent tests. For radiological markers of CSVD, we performed a confirmatory analysis using individual-level data in UK Biobank:

#### Two-Sample MR Using Summary Statistics

We assessed the impact of risk factor–associated SNPs on each CSVD phenotype using MR approaches. Our primary analysis used an inverse-variance weighted meta-analysis approach (conventional MR). We then performed secondary analyses using weighted median and penalized weighted median approaches. We assessed the potential role of directional pleiotropy by testing if the intercept from MR-Egger regression was significantly different from zero.

As sensitivity analysis, we performed a look-up of all the SNPs used in our study in Phenoscanner (http://www.phenoscanner.medschl.cam.ac.uk/phenoscanner) to evaluate whether these SNPs were associated with other traits at genome-wide significance level which may affect our results. We found 8 SNPs related to T2D (rs2925979, rs2943640, rs3794991, rs3923113, rs429358, rs459193, rs635634, and rs780094), which were also associated with lipids and kidney function, as well as 4 SNPs for fasting glucose (rs174550, rs17762454, rs780094, and rs983309) and 9 SNPs for fasting insulin (rs10195252, rs1530559, rs2126259, rs2745353, rs2943645, rs3822072, rs459193, rs731839, and rs780094). We then reassessed the results after excluding these SNPs. Additionally, for significant findings, we assessed the potential that the association was mediated by body mass index (BMI) by performing a sensitivity analysis, removing 8 SNPs, which are also associated with BMI at genome-wide significance (rs2943640, rs11671664, rs12970134, rs8050136, rs10146997, rs5215, and rs7903146). All analyses were performed using the Mendelian Randomization and gtx libraries in R version 3.3.2 (https://www.R-project.org/).

#### Two-Sample MR Using Genetic Risk Scores on Individual-Level Data

For each of the 84 SNPs associated with T2D, we constructed a genetic risk score for each individual in UK Biobank by multiplying the log of the odds ratio (OR) for association with T2D by the number of risk alleles and summing this value for each individual. We then used a linear regression model to evaluate the effect of the genetic score on WMH, FA, and MD, with adjustment for genotyping batch, age, sex, BMI, blood pressure, and ancestry-informative principal components to control for these potential confounding factors.

We also assessed their associations by constructing quartiles of the genetic score and calculated the ORs and 95% confidence interval (CI) for the score quartiles using quantile 1 as a reference, thus quantile 2 versus quantile 1, quantile 3 versus quantile 1, and quantile 4 versus quantile 1.

## Results

### MR Results: Associations of T2D With CSVD Phenotypes

Conventional MR estimates for the effect of T2D on clinical outcomes associated with CSVD (lacunar stroke, ICH, deep ICH, and lobar ICH) and radiological markers of CSVD (WMH, FA, and MD) are displayed in the Figure and Table 2.

**Table 2.**
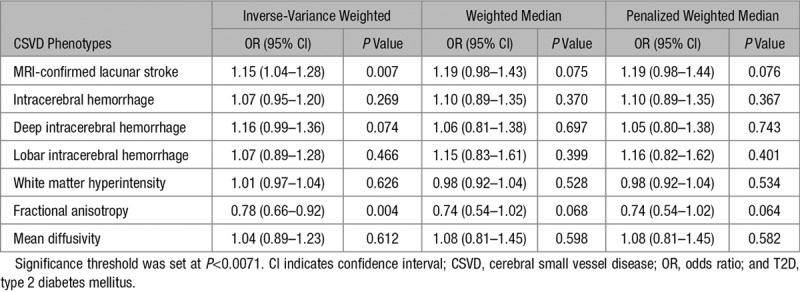
Mendelian Randomization Estimates for the Effect of T2D on CSVD Phenotypes Using Inverse-Variance Weighted, Weighted Median, and Penalized Weighted Median Methods

**Figure.**
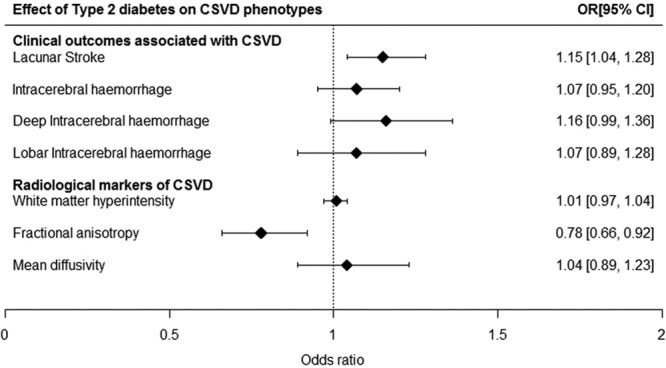
Mendelian randomization estimates for the effect of type 2 diabetes mellitus (T2D) on cerebral small vessel disease (CSVD) phenotypes. Analyses were performed with conventional Mendelian randomization analysis (inverse-variance weighted method). CI indicates confidence interval; and OR, odds ratio.

T2D was associated with higher risk of lacunar stroke (OR, 1.15; 95% CI, 1.04–1.28; *P*=0.007) and lower mean FA (OR, 0.78; 95% CI, 0.66–0.92; *P*=0.004). Conversely, we did not find any significant associations of T2D with WMH volume (OR, 1.01; 95% CI, 0.97–1.04; *P*=0.626) and higher mean MD (OR, 1.04; 95% CI, 0.89–1.23; *P*=0.612). Regarding the effect of T2D on ICH, lobar ICH, and deep ICH, none were found to be significant (ICH risk: OR, 1.07; 95% CI, 0.95–1.20; *P*=0.269; lobar ICH risk: OR, 1.07; 95% CI, 0.89–1.28; *P*=0.466; deep ICH risk: OR, 1.16; 95% CI, 0.99–1.36; *P*=0.074). Although nonsignificant, the odds ratio for association with deep ICH was near significant with an OR and direction of effect similar to that seen for lacunar stroke.

Weighted median and penalized median weighted analysis yielded similar effect estimates of T2D on lacunar stroke and FA, but the CIs were wide, so were not significant (Table 2). To assess the robustness and consistency of the results, we also conducted a sensitivity analysis by excluding the SNPs associated with lipids and kidney function at genome-wide significance level. In this sensitivity analysis, the associations of T2D with lacunar stroke and FA remained significant (Table III in the online-only Data Supplement). To assess whether the results were influence by BMI, we repeated the analyses for T2D and lacunar stroke as well as T2D and FA, both of which showed only minor differences after removing the SNPs (lacunar stroke: OR, 1.14; 95% CI, 1.01–1.29; *P*=0.028 and FA: OR, 0.78; 95% CI, 0.64–0.94; *P*=0.0095).

MR-Egger regression showed no evidence of directional pleiotropy for the effects of T2D on lacunar stroke (intercept=0.008; *P*=0.515), ICH (intercept=0.009; *P*=0.528), deep ICH (intercept=0.022; *P*=0.218), lobar ICH (intercept=0.014; *P*=0.539), WMH (intercept=0.003; *P*=0.518), FA (intercept=−0.003; *P*=0.901), and MD (intercept=0.004; *P*=0.869).

### Genetic Risk Score Analyses: Effects of T2D on WMH, FA, and MD

Results of the T2D genetic risk score on WMH, FA, and MD are presented in Table 3. The genetic score including 84 T2D-associated SNPs was significantly associated with FA (OR, 0.63; 95% CI, 0.45–0.89; *P*=0.008) after adjustment for genotyping batch, age, sex, BMI, blood pressure, and ancestry-informative principal components. We note that this result is significant when correcting for the 3 phenotypes studied in this secondary analysis but does not reach *P*<0.0071, the threshold used in our primary analysis. Conversely, the risk score was not significantly associated with WMH (OR, 1.01; 95% CI, 0.94–1.09; *P*=0.727) or MD (OR, 1.09; 95% CI, 0.78–1.52; *P*=0.613).

**Table 3.**
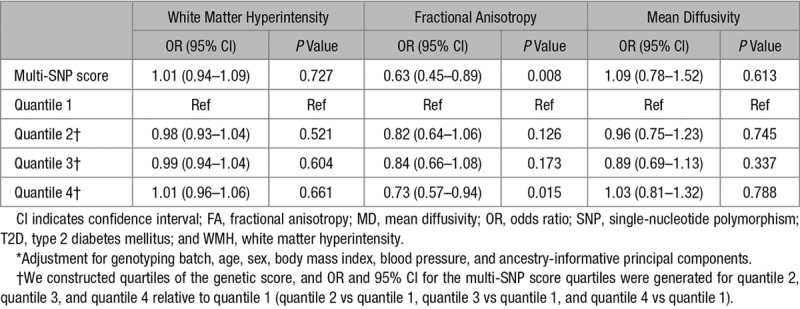
Association of the T2D-Related SNPs With WMH, FA, and MD in UK Biobank Using Linear Regression*

Stratifying by quartiles of the genetic score, we only found a nominally significant effect of quantile 4 compared with quantile 1 of the score on FA (OR, 0.73; 95% CI, 0.57–0.94; *P*=0.015).Odds ratios±SE for WMH, FA, and MD based on the quartiles of the genetic score were plotted (Figures I through III in the online-only Data Supplement).

### Associations of Fasting Glucose and Insulin With CSVD Phenotypes

Fasting glucose and insulin were not associated with any CSVD phenotypes (lacunar stroke, ICH, deep ICH, lobar ICH, WMH, FA, and MD) in the present study using inverse-variance weighted, weighted median, or MR-Egger regression methods (Table II in the online-only Data Supplement).

## Discussion

Prevention and management of CSVD is limited by our understanding of causal factors underlying the disease process. T2D is an established risk factor for CSVD, but a causal relationship is yet to be determined, and its impact on different CSVD phenotypes is not well understood. Using genetic data via an MR approach, we assessed the causal relationship between T2D and different CSVD phenotypes. Our primary analysis showed that a genetic predisposition to T2D was related to lacunar stroke and FA. We performed secondary analyses using an alternative weighted median method, which showed similar effects but wider CIs. Evidence indicating that the associations are robust is provided by the fact that (1) the results remained in sensitivity analyses removing pleiotropic SNPs and (2) associations with FA were significant when performing an alternative analysis based on individual-level data in UK Biobank, in which we were able to adjust for potential confounding factors.

The results were consistent with previous observational studies showing a positive association of T2D with risk of lacunar stroke^6,7^ and an MR analysis using small artery occlusion strokes based on the TOAST classification (Trial of ORG 10172 in Acute Stroke Treatment).^25^ However, the OR values of the relationship between T2D and lacunar stroke from a meta-analysis published in 2006^6^ ranged from 1.3 to 2.2 in different cohorts, which were larger than our MR analyses (OR, 1.15; 95% CI, 1.04–1.28). This may be explained by the fact that previous observational epidemiological associations may have been influenced by potentially important confounders such as sex, blood pressure, and dietary factors. It should also be noted that the OR in our study refers to the increased risk associated with genetic markers related to T2D. Therefore, as these do not capture the total variance associated with T2D, the estimate is likely to be smaller than those from epidemiological studies.

Studies of the association between T2D and pathogenic subtypes of ICH are limited, but a recent meta-analysis^9^ included 19 case–control studies has reported that hemorrhagic stroke was 1.23-fold more prevalent in patients with diabetes mellitus, whereas the association was not observed in 3 population-based cohort studies.^26–28^ Our study using an MR approach showed no significant associations of T2D on total ICH risk, and on different location of ICH risk (lobar and deep ICH^29,30^). Although not significant, the effect of T2D on deep ICH was similar to that seen in lacunar stroke, which is consistent with the idea that deep ICH is associated with CSVD. However, we had limited power because of the small sample size for ICH, and this needs to be studied in larger data sets.

To better assess the causality of the associations between T2D and cerebral white matter injury, we used macrostructural (WMH volume) and microstructural (FA and MD) brain magnetic resonance imaging measures for the total brain. First, both MR approach and genetic score analyses did not show any significant associations between T2D and WMH volume, which was similar to the results from 2 recent reviews^6,7^ showing uncertainty for the effect of T2D on WMH. In addition, data from a randomized controlled trial study^31^ found that intensive control of diabetes mellitus (hemoglobin A1c <6.0%) did not impede WMH progression and conversely caused more serious WMH for unclear reasons.

Second, FA and MD are 2 commonly used diffusion tensor imaging indices, which are highly sensitive to subtle white matter changes.^32^ A few observational studies^33–36^ have shown a decreased FA and increased MD in T2D patients compared with controls, and the results were largely independent of WMH volume, which suggested that microstructural integrity damage likely precedes WMH, and diffusion tensor imaging indices maybe early markers for brain damage in patients with T2D. The results from the current study partly confirmed the findings from these aforementioned observational studies. We found the significant association between T2D and FA using MR methods, which also remained consistent in genetic score analyses. Surprisingly, T2D was not significantly associated with increased MD, and the reason for this discrepancy is unclear. It has been suggested that FA decrease maybe modulated more directly by myelin alterations, whereas MD is more sensitive to cellularity, edema, and necrosis.^37^ Our results might, therefore, point to demyelination being an important factor in T2D patients.

Strengths of our MR analysis include the use of multiple T2D SNPs, which enable us to construct a polygenic score to increase the precision of the estimates. In combination, the SNPs explained between 5% and 10% of the variance of T2D. Ours is the first MR study to investigate the relationship between T2D and CSVD phenotypes, and the design of MR study can prevent reverse causation and potential confounding factors,^13,14^ such as lifestyle and dietary preference. However, there are some limitations in our MR study. First, in some of our MR analyses, the sample size was relatively small, which resulted in limited statistical power in the respective analyses, especially for the ICH analysis stratified by hemorrhage location. Second, effects of the genetic variants on T2D were obtained largely from European populations, and all the subjects included in our study were Europeans. Therefore, the results may not be generalizable to other populations. Additionally, we note that differences in baseline characteristics between the T2D and CSVD populations might have subtle influences on the effect estimates, meaning that OR values given here should be interpreted with this caveat. Limitations of MR include the potential for residual pleiotropy that could have influenced the results when T2D-associated SNPs also influence other traits. We note that this is often challenging to rule out with absolute certainty.^38^ One possible pleiotropic pathway in this analysis is through elevated BMI. From the results presented here, we cannot rule out partial mediation of the genetic effects through this pathway. Finally, our significant association of T2D with lacunar stroke and FA was not confirmed after conducting weighted median and penalized weighted median methods. However, the weighted median approaches showed a similar effect size. MR studies with larger samples are, therefore, needed to confirm this result in the future.

## Conclusions

Our MR study is consistent with a causal association between T2D and CSVD. In particular, we found evidence of associations with lacunar stroke and FA. Further MR studies with larger sample sizes are required to determine this with more certainty and to rule out associations with other CSVD phenotypes.

## Acknowledgments

The Genetics of magnetic resonance imaging–confirmed lacunar stroke project contains samples derived from the SIGN-NINDS study, the WTCCC2 stroke study, and DNA lacunar. The SiGN study was funded by a cooperative agreement grant from the US National Institute of Neurological Disorders and Stroke, National Institutes of Health (U01 NS069208). Collection of the UK Young Lacunar Stroke DNA Study (DNA Lacunar) was primarily supported by the Wellcome Trust (WT072952) with additional support from the Stroke Association (TSA 2010/01). Genotyping of the DNA Lacunar samples was supported by a Stroke Association Grant (TSA 2013/01). The principal funding for the WTCCC2 stroke study was provided by the Wellcome Trust, as part of the Wellcome Trust Case Control Consortium 2 project (085475/B/08/Z and 085475/Z/08/Z and WT084724MA). The present analyses were conducted under UK Biobank application number 19463.

## Sources of Funding

Dr J. Liu was sponsored by China scholarship Council. Dr M. Liu is supported by Major International (Regional) Joint Research Project, National Natural Science Foundation of China (grant number: 81620108009). This work was supported by a British Heart Foundation Programme Grant (RG/16/4/32218). H. S. Markus is supported by a National Institute for Health Research (NIHR) Senior Investigator award, and his work is supported by the Cambridge Universities NIHR Comprehensive Biomedical Research Centre.

## Disclosures

None.

## Supplementary Material
