# Supplementary material for: Causal Impact of Type 2 Diabetes Mellitus on Cerebral Small Vessel Disease: A Mendelian Randomization Analysis
Source: Stroke. 2018 Apr 23;49(6):1325–31. doi: 10.1161/STROKEAHA.117.020536 (PMC5976219; doi:10.1161/STROKEAHA.117.020536)
Supplement: Supplementary file 1 [file str-49-1325-s001.pdf]

SUPPLEMENTAL MATERIAL

Table I. Single nucleotide polymorphism used as instrumental variables in the Mendelian randomization analyses of type 2 diabetes, fasting glucose, and fasting insulin

SNP	Closest gene	Risk factor	Effect allele	Risk factor Beta
rs10146997	NRXN3/DIO2	T2D	G	0.068
rs10193447	*BCL11A*	T2D	T	0.068
rs10238625	*DGKB*	T2D	A	0.068
rs10507349	RNF6	T2D	A	-0.056
rs1061810	*HSD17B12*	T2D	A	0.077
rs10811661	CDKN2A/2B	T2D	C	-0.156
rs10830963	*MTNR1B*	T2D	G	0.094
rs10842994	KLHDC5	T2D	T	-0.081
rs1111875	HHEX	T2D	T	-0.107
rs11123406	BCL2L11	T2D	C	-0.049
rs111669836	*MAP3K11*	T2D	A	0.068
rs11257655	CDC123	T2D	T	0.077
rs11671664	GIPR	T2D	A	0.074
rs11708067	*ADCY5*	T2D	G	-0.104
rs11759026	*CENPW*	T2D	G	0.095
rs11787792	*GPSM1*	T2D	A	0.130
rs1182436	*MNX1*	T2D	C	0.077
rs12970134	MC4R	T2D	A	0.071
rs13266634	SLC30A8	T2D	T	-0.111
rs13292136	TLE4	T2D	T	-0.129
rs1359790	SPRY2	T2D	A	-0.077
rs1552224	ARAP1	T2D	C	-0.101
rs163184	KCNQ1 [1]	T2D	G	0.079
rs17106184	FAF1	T2D	A	-0.090
rs17168486	DGKB	T2D	T	0.093
rs1801282	PPARG	T2D	G	-0.113
rs2023681	*MTMR3/HORMAD2*	T2D	G	0.122
rs2050188	C6orf10	T2D	T	0.051
rs2237897	*KCNQ1*	T2D	C	0.223
rs2292626	*PLEKHA1*	T2D	C	0.086
rs231360	*KCNQ1*	T2D	T	0.077
rs2706785	TMEM155	T2D	G	0.128
rs2796441	TLE1	T2D	A	-0.064
rs2925979	*CMIP*	T2D	T	0.077
rs2943640	IRS1	T2D	C	0.082
rs329122	JADE2	T2D	A	0.045
rs340874	*PROX1*	T2D	C	0.060
rs35352848	*UBE2E2*	T2D	T	0.086

rs3794991	GATAD2A|CILP2	T2D	T	0.130
rs3821943	*WFS1*	T2D	T	0.095
rs3923113	GRB14	T2D	C	-0.069
rs4238013	*CCND2*	T2D	C	0.095
rs429358	*APOE*	T2D	T	0.122
rs4402960	*IGF2BP2*	T2D	T	0.140
rs4457053	ZBED3	T2D	A	-0.080
rs459193	ANKRD55	T2D	G	0.078
rs4774420	*C2CD4A*	T2D	C	0.077
rs4812829	HNF4A	T2D	A	0.053
rs516946	*ANK1*	T2D	C	0.078
rs5215	KCNJ11	T2D	T	-0.075
rs576674	KL	T2D	A	-0.073
rs60780116	*ACSL1*	T2D	T	0.086
rs622217	SLC22A3	T2D	C	-0.053
rs635634	*ABO*	T2D	T	0.077
rs6813195	TMEM154	T2D	T	-0.064
rs6918311	*SLC35D3*	T2D	A	0.068
rs702634	ARL15	T2D	A	0.050
rs7041847	GLIS3	T2D	G	-0.042
rs7111341	MIR4686	T2D	T	0.051
rs7224685	*ZZEF1*	T2D	T	0.068
rs7428936	*ADAMTS9*	T2D	T	0.068
rs7451008	*CDKAL1*	T2D	C	0.174
rs756852	*KCNQ1*	T2D	G	0.086
rs757209	*HNF1B (TCF2)*	T2D	G	0.086
rs7578597	THADA	T2D	C	-0.138
rs7674212	SLC9B2	T2D	T	-0.063
rs780094	GCKR	T2D	C	0.055
rs78761021	*GLP2R*	T2D	G	0.068
rs7903146	*TCF7L2*	T2D	T	0.329
rs79349575	*GIP*	T2D	A	0.068
rs7957197	HNF1A	T2D	A	-0.077
rs7985179	MIR17HG	T2D	A	-0.089
rs8042680	PRC1	T2D	A	0.063
rs8050136	FTO	T2D	A	0.107
rs8056814	*BCAR1*	T2D	G	0.148
rs810517	*ZMIZ1*	T2D	C	0.086
rs864745	JAZF1	T2D	C	-0.085
rs9271774	*HLA-DQA1*	T2D	C	0.095
rs944801	CDKN2B-AS1	T2D	C	0.071
rs9505118	SSR1	T2D	G	-0.063
rs952471	*HMG20A*	T2D	G	0.077
rs9648716	BRAF	T2D	T	0.076
rs9687833	*ANKRD55*	T2D	A	0.095
rs9940149	ITFG3|HCCA2	T2D	A	-0.062

rs10747083	P2RX2	Fasting glucose	A	0.016
rs10811661	CDKN2B	Fasting glucose	T	0.027
rs10830963	MTNR1B	Fasting glucose	G	0.077
rs10885122	ADRA2A	Fasting glucose	G	0.022
rs11071657	VPS13C/C2CD4 A/B	Fasting glucose	A	0.006
rs11558471	SLC30A8	Fasting glucose	G	0.029
rs11603334	ARAP1	Fasting glucose	G	0.019
rs11605924	CRY2	Fasting glucose	A	0.019
rs11619319	PDX1	Fasting glucose	G	0.021
rs11708067	ADCY5	Fasting glucose	A	0.021
rs11715915	AMT	Fasting glucose	C	0.013
rs11920090	SLC2A2	Fasting glucose	T	0.028
rs16913693	IKBKAP	Fasting glucose	T	0.047
rs174550	FADS1	Fasting glucose	T	0.018
rs17762454	RREB1	Fasting glucose	T	0.012
rs2191349	DGKB/TMEM19 5	Fasting glucose	T	0.029
rs2302593	GIPR	Fasting glucose	C	0.014
rs2657879	GLS2	Fasting glucose	G	0.011
rs340874	PROX1	Fasting glucose	C	0.010
rs3783347	WARS	Fasting glucose	G	0.015
rs3829109	LOC728489	Fasting glucose	G	0.018
rs4506565	TCF7L2	Fasting glucose	T	0.019
rs4607517	GCK	Fasting glucose	A	0.055
rs4869272	PCSK1	Fasting glucose	T	0.018
rs560887	G6PC2	Fasting glucose	C	0.070
rs576674	KL	Fasting glucose	G	0.019
rs6072275	TOP1	Fasting glucose	A	0.016
rs6113722	FOXA2	Fasting glucose	G	0.038
rs6943153	GRB10	Fasting glucose	T	0.016
rs7651090	IGF2BP2	Fasting glucose	G	0.014
rs7708285	ZBED3	Fasting glucose	G	0.009
rs780094	GCKR	Fasting glucose	C	0.028
rs7867224	GLIS3	Fasting glucose	A	0.014
rs7944584	MADD	Fasting glucose	A	0.023
rs9368222	CDKAL1	Fasting glucose	A	0.016
rs983309	PPP1R3B	Fasting glucose	T	0.025
rs10195252	GRB14	Fasting insulin	T	0.018
rs1167800	HIP1	Fasting insulin	A	0.015

rs1530559	YSK4	Fasting insulin	A	0.013
rs17036328	PPARG	Fasting insulin	T	0.014
rs2126259	PPP1R3B	Fasting insulin	T	0.031
rs2745353	RSPO3	Fasting insulin	T	0.015
rs2943645	IRS1	Fasting insulin	T	0.016
rs3822072	FAM13A1	Fasting insulin	A	0.010
rs459193	ANKRD55	Fasting insulin	G	0.019
rs4846565	LYPLAL1	Fasting insulin	G	0.015
rs4865796	ARL15	Fasting insulin	A	0.016
rs6822892	PDGFC	Fasting insulin	A	0.010
rs6912327	C6orf107	Fasting insulin	T	0.015
rs731839	PEPD	Fasting insulin	G	0.017
rs780094	GCKR	Fasting insulin	C	0.022
rs7903146	TCF7L2	Fasting insulin	C	0.022
rs860598	IGF1	Fasting insulin	A	0.012
rs974801	TET2	Fasting insulin	G	0.016

Table II. Mendelian randomization estimates for the effect of Fasting glucose, and insulin on CSVD phenotypes using inverse-variance weighted, weighted median and penalized weighted median methods

	Lacunar stroke OR(95%CI)	P	ICH OR(95%CI)	P	Deep ICH OR(95%CI)	P	Lobar ICH OR(95%CI)	P	WMH OR(95%CI)	P	FA OR(95%CI)	P	MD OR(95%CI)	P
**Inverse-variance weighted**														
Fasting Glucose	1.10(0.63-1.90)	0.744	0.65(0.36-1.20)	0.167	0.75(0.32-1.74)	0.504	0.71(0.28-1.78)	0.464	1.16(0.97-1.40)	0.099	0.44(0.18-1.08)	0.073	1.85(0.76-4.52)	0.177
Fasting Insulin	1.52(0.45-5.08)	0.500	0.48(0.12-1.86)	0.288	0.18(0.03-1.05)	0.056	0.41(0.06-2.98)	0.378	1.04(0.69-1.54)	0.843	1.61(0.23-11.32)	0.635	0.67(0.09-4.74)	0.685
**Weighted median**														
Fasting Glucose	1.14(0.53-2.44)	0.733	0.60(0.25-1.44)	0.249	0.47(0.14-1.54)	0.213	2.10(0.53-8.40)	0.294	1.01(0.78-1.32)	0.934	0.62(0.16-2.37)	0.484	1.99(0.49-8.18)	0.338
Fasting Insulin	2.11(0.39-11.39)	0.383	0.18(0.03-1.24)	0.081	0.19(0.02-2.28)	0.192	0.13(0.01-1.86)	0.133	1.10(0.64-1.91)	0.728	0.79(0.05-13.39)	0.868	0.48(0.03-8.17)	0.608
**Penalized weighted median**														
Fasting Glucose	1.16(0.54-2.48)	0.696	0.60(0.25-1.44)	0.249	0.47(0.14-1.54)	0.211	2.16(0.54-8.55)	0.275	1.01(0.78-1.31)	0.946	0.62(0.16-2.37)	0.484	2.01(0.46-8.77)	0.355
Fasting Insulin	2.38(0.44-12.89)	0.316	0.17(0.02-1.21)	0.077	0.17(0.01-2.04)	0.163	0.12(0.01-1.72)	0.119	1.10(0.64-1.91)	0.728	0.69(0.04-12.01)	0.802	0.38(0.02-6.46)	0.499

Abbreviations:CSVD=cerebral small vessel disease; CI = confidence interval; OR = odds ratio,ICH=Intracerebral haemorrhage, WMH=White matter hyperintensity, FA=Fractional anisotropy, MD=Mean diffusivity.

*MR-Egger regression estimates could not be identified because the SNPs had similar magnitudes of association with fasting insulin.

Table III. Mendelian randomization estimates for the effect of each risk factor on CSVD phenotypes using inverse-variance weighted for sensitivity analyses*

	**Lacunar stroke**		**ICH**		**Deep ICH**		**Lobar ICH**		**WMH**		**FA**		**MD**	
	**OR(95%CI)**	**P**	**OR(95%CI)**	**P**	**OR(95%CI)**	**P**	**OR(95%CI)**	**P**	**OR(95%CI)**	**P**	**OR(95%CI)**	**P**	**OR(95%CI)**	**P**
Type 2 diabetes	1.17(1.05-1.31)	0.004	1.08(0.96-1.22)	0.193	1.16(0.98-1.38)	0.081	1.10(0.92-1.33)	0.300	1.02(0.98-1.06)	0.264	0.77(0.64-0.91)	0.002	1.08(0.91-1.27)	0.397
Fasting Glucose	1.03(0.58-1.84)	0.908	0.64(0.34-1.20)	0.166	0.87(0.36-2.08)	0.747	0.80(0.04-17.86)	0.889	1.17(0.97-1.41)	0.106	0.40(0.16-1.00)	0.051	1.75(0.69-4.42)	0.238
Fasting Insulin	5.68(0.86-37.46)	0.071	0.86(0.11-6.82)	0.889	0.92(0.05-15.41)	0.953	0.81(0.04-17.87)	0.889	1.23(0.66-2.28)	0.509	2.62(0.13-54.20)	0.534	1.54(0.07-32.28)	0.780

Abbreviations:CSVD=cerebral small vessel disease; CI = confidence interval; OR = odds ratio, ICH=Intracerebral haemorrhage, WMH=White matter hyperintensity, FA=Fractional anisotropy, MD=Mean diffusivity.

*Excluded the SNPs associated with lipids and kidney function.

Table IV Studies included in the MRI-confirmed lacunar stroke collaboration

Cohorts	Cases	Controls
DNA LACUNAR	917	
GENESIS (SR/ADDS)	299	
PRESERVE	46	
UK-WTCCC2	250	
GERMANY-WTCCC2	37	
MILANO	9	
ASGC	23	
SIGN:BRAINS	5	
SIGN:GEOS	4	
SIGN:GCNKSS	27	
SIGN:MIAMISR	13	
SIGN:GASROS	27	
SIGN:ISGS	28	
SIGN:KRAKOW	7	
SIGN:LEUVEN	45	
SIGN:BASICMAR	36	
SIGN:SAHLSIS	31	
SIGN:SPS3_EUR	345	
SIGN:GRAZ	42	
1958 BIRTH COHORT, NATIONAL BLOOD SERVICE		5175
DNA LACUNAR		968
KORA		797
SIGN:HRS		9286
SIGN:GRAZ		816
SIGN:LEUVEN		453
SIGN:KRAKOW		716
SIGN:ADHD		411
SIGN:MALMO		1362
SIGN:OAI		3201
SIGN:GEOS		519
SIGN:ASGS		1200
SIGN:HABC		1586
SIGN:INMA		807
**TOTAL EUROPEAN**	**2191**	**27297**

Standard thorough quality control was performed on each dataset separately. The data were then aligned to the forward strand and imputed to the HRC reference panel. SNPs with MAF<0.01 or INFO<0.5 were then removed and association analysis was carried out using RVTESTS including age, sex, study group, and the first 10 principal components. The inflation of test statistics (λ) was equal to the inflation expected for the sample size.

Figure I. Odds ratios ± standard error for WMH (logWMH volume) based on multi-SNP score (Quantile2 vs Quantile 1, Quantile 3 vs Quantile 1 and Quantile 4 vs Quantile 1)

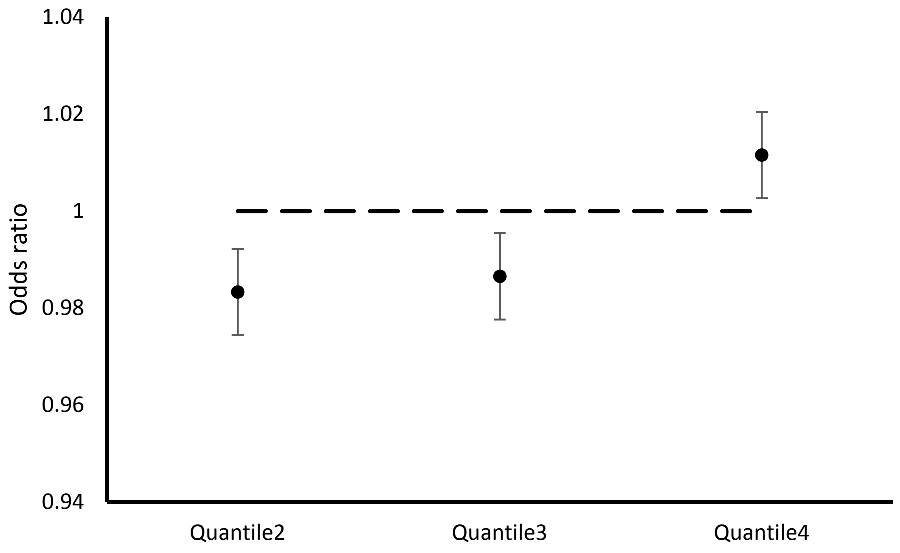

Figure II.Odds ratios ± standard error for FA based on multi-SNP score(Quantile2 vs Quantile 1, Quantile 3 vs Quantile 1 and Quantile 4 vs Quantile 1)

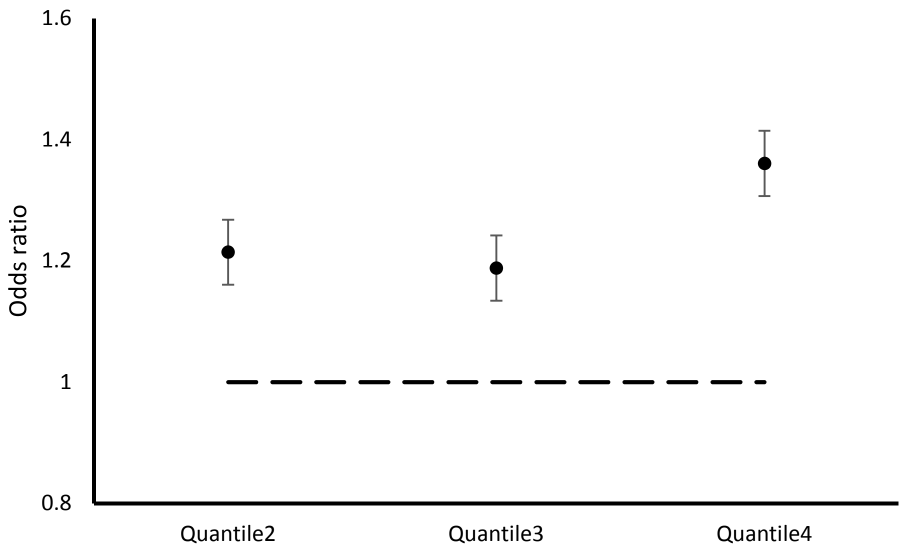

Figure III. Odds ratios ± standard error for MD based on multi-SNP score (Quantile2 vs Quantile 1, Quantile 3 vs Quantile 1 and Quantile 4 vs Quantile 1)

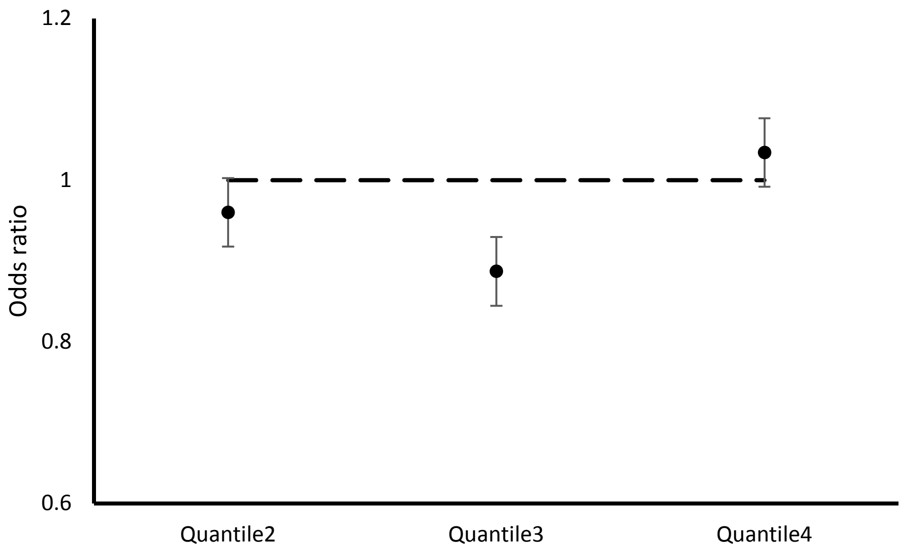

Supplementary methods

UK Biobank genome-wide association study of FA, MD, and WMH

Study population and ethical approval

UK Biobank is a prospective study that recruited 500,000 community-dwelling participants from across the UK between 2006 and 2010, aged 40–69 years (http://www.ukbiobank.ac.uk). The study collects extensive data from questionnaires, interviews, health records, physical measures, biological samples and imaging.

A subset of the participants also underwent brain MRI. In the present study we used the second release of MRI data, which included 9,045 subjects who underwent brain MRI, on average 6.6 years (SD 1.0 years) after initial recruitment at mean age 55.5 years (SD 7.4 years) and had usable T2 FLAIR or DTI images. Patients with a baseline diagnosis of stroke (ICD-9/-10 or self-report or health-record linkage), multiple sclerosis, Parkinson's disease, dementia, any other neurodegenerative problem or no genetic data were excluded. A further three participants with consistently extreme outlying tract-averaged water diffusion biomarker values were removed following visual inspection of the data by the authors, leaving 8,568 individuals for the current analysis.

UK Biobank received ethical approval from the research ethics committee (REC reference 11/NW/0382). All participants provided informed consent to participate. The present analyses were conducted under UK Biobank application number 19463.

MRI

Procedures for brain imaging acquisition and initial quality check have been described previously and are available on the UK Biobank website (Brain Imaging Documentation V1.3, http://www.ukbiobank.ac.uk).[1]

In brief, all brain MRI data were acquired on a single standard Siemens Skyra 3T scanner (Siemens Medical Solutions, Germany) using the standard Siemens 32-channel RF receiver head coil. Sagittal T1-weighted scans were acquired using a 3D magnetization-prepared rapid acquisition gradient-echo (MPRAGE) sequence (resolution 1 x 1 x 1 mm, field of view 208 x 256 x 256, TI/TR=880/2000 ms). Sagittal T2-weighted fluid attenuated inversion recovery (FLAIR) scans were obtained using a 3D SPACE sequence (resolution 1.05 x 1.0 x 1.0 mm, field of view 192 x 256 x 256, TI/TR=1800/5000 ms). DTI scans were acquired with a spin-echo echo-planar imaging sequence and multi-shell acquisition (b0 = 0 s/mm^{-2}, b = 1,000 s/mm^{-2}, b = 2,000 s/mm^{-2}, 100 distinct diffusion-encoding directions, 2 mm isotropic voxels, field of view 104 x 104 x 72).

White matter hyperintensities

White matter hyperintensities (WMH) were automatically segmented using the combined T1 and T2-FLAIR data as input in the BIANCA tool (Brain Intensity AbNormality Classification Algorithm).[2]BIANCA is a fully-automated supervised method for WMH detection, based on the k-nearest neighbour algorithm, which gives the probability per voxel of being WMH. The total WMH volume was calculated from the voxels exceeding a probability of 0.9 of being WMH and located within a white matter mask. Obtained values were adjusted for the total intracranial volume and log transformed because of their skewed distribution.

Fractional anisotropy and mean diffusivity

Following gradient distortion correction and further correction for head movement and eddy currents, diffusion tensors and scalar diffusion parameters (ie, FA and MD) were calculated by feeding the b=1000 shell (50 directions) into DTIFIT within the FSL software.[3] The FA maps were fed into tract-based spatial statistics (TBSS) which aligns the FA map onto a standard-space white matter skeleton. The resulting standard-space warp is applied to all other DTI outputs. MD images were projected onto the skeleton, using the FA derived projection parameters. Subsequently the skeletonised images were averaged across a set of 48 standard-space tract masks, similar to the processing applied in the ENIGMA project (http://enigma.ini.usc.edu/protocols/dti-protocols).[4,5] Principal component analysis (PCA) was applied on the 48 tracts to extract a latent measure. The first principal component of FA (FA.PC1) and MD (MD.PC1) was used in subsequent analyses as dependent variable.

Genetic data

We used the June 2017 release of the imputed genetic data from UK Biobank (downloaded on June 3, 2017). Details of the design of the arrays, sample processing and stringent quality control have been described elsewhere.[6]In brief, two closely related arrays from Affymetrix, the UK BiLEVE Axiom array (9.9% of individuals) and the UK Biobank Axiom array, were used to genotype approximately 805,426 markers with good genome-wide coverage. Phasing was performed using SHAPEIT3 and imputation to a merged HRC reference panel (39,131,578 autosomal SNPs) and UK10K & 1000 Genomes Phase 3 panel was carried out using the IMPUTE4 package.[6–8] Imputed genotypes were available for 487,442 individuals in this study. From the resulting dataset, we excluded (1) individuals that did not segregate with European samples based on PCA analysis, (2) individuals with high levels heterozygosity and missingness (>5%), (3) individuals whose reported sex was inconsistent with sex inferred from the genetic data. In addition, only SNPs imputed from the HRC panel were included in this analysis.

Statistical analysis

In this analysis, we first subset the genetic data on the individuals that also had MRI imaging data. We performed a genome-wide association study of FA, MD and log(WMH), using SNPTEST v2.5.4-beta3 including age at MRI, sex, body mass index (BMI), genotyping batch, and the first 10 ancestry informative principal components as covariates.

Intracerebral Haemorrhage Meta-analysis

Contributing Studies

1. Cambridge ICH Genetics Study.

Intracerebral Haemorrhage cases were recruited based on hospital admissions at St. George's Hospital, London and Addenbrooke's Hospital, Cambridge as part of the St. George's Stroke Register and GENESIS studiesbetween 2002-2012. All cases were confirmed radiologically.

Unrelated Caucasian controls, free of clinical cerebrovascular disease, were obtained by random sampling, stratified for age and sex, from general practice lists from the same geographical location as the patients. All patients and controls underwent a standardized clinical assessment and completed a standardized study questionnaire.

The genetic dataset, which included other individuals not eligible for this analysis, was genotyped on the Illumina HumanCoreExome array. SNPs were excluded with MAF<0.01, genotype missingness>3%, HWE p<1e-6 in controls, strand ambiguity (A/T or C/G) or evidence of differential missingness by case-control status (p<0.05). Individuals were excluded if they had missingness>3%, excess or reduced heterozygosity, showed evidence of relatedness with another individual (pi-hat>0.1875), or failed a "sex-check" in PLINK. EIGENSTRAT was used to remove non-caucasian individuals, and was then repeated to calculate ancestry-informative principal components. The remaining 269,691 autosomal SNPs and 2,603 individuals were then imputed to the haplotype reference consortium build 2016.1 using the Michigan Imputation Server.[7]Post-imputation, SNPs were removed with poor imputation quality (INFO<0.5) or low minor allele frequency (MAF<.005).

Analysis of the relationship between genomewide SNP dosages and ICH was performed using rvtests[9],adjusting for age, sex and ancestry informative-principal components.

2. Intracerebral Haemorrhage Genetics Collaboration

The intracerebral Haemorrhage Genetics Collaboration is composed of 1,545 cases and 1481 controls from studies from the United States and Europe. Full details of the population, genotyping and imputation are available elsewhere.[10]

3. UK Biobank ICH GWAS

We used the June 2017 release of the imputed genetic data from UK Biobank (downloaded on June 3, 2017). Details of the design of the arrays, sample processing and stringent quality control have been described elsewhere.[6]In brief, two closely related arrays from Affymetrix, the UK BiLEVE Axiom array (9.9% of individuals) and the UK Biobank Axiom array, were used to genotype approximately 805,426 markers with good genome-wide coverage. Phasing was performed using SHAPEIT3 and imputation to a merged HRC reference panel (39,131,578 autosomal SNPs) and UK10K & 1000 Genomes Phase 3 panel was carried out using the IMPUTE4 package.[6–8] Imputed genotypes were available for 487,442 individuals in this study.

From the resulting dataset, we excluded (1) individuals not designated as 'White British' based on central PCA analysis, (2) individuals with high levels heterozygosity and missingness (>5%), (3) individuals whose reported sex was inconsistent with sex inferred from the genetic data. In addition, only SNPs imputed from the HRC panel were included in this analysis.Post-imputation, SNPs were removed with poor imputation quality (INFO<0.5) or low minor allele frequency (MAF<.005).

We used algorithmically defined intracerebral haemorrhage as our outcome. We derived a set of controls at a 10:1 ratio based on propensity score matching on age, sex and ancestry-informative principal components.

Analysis of the association between genome-wide genotype dosages and the resulting 575 cases and 5750 controls was performing using rvtests[9]adjusting for age, sex, and ancestry-informative principal components.

We note that haemorrhage location information was not available in UK Biobank, meaning subgroup (deep or lobar) analyses could therefore not be performed.

Meta-analysis Methods

The contributing datasets were analysed using a fixed-effects inverse variance weighted method using METAL.[11] Genomic Control was used to control for any residual inflation. Only SNPs present in all 3 contributing datasets were considered in this analysis.

References

1. Alfaro-Almagro F, Jenkinson M, Bangerter NK, Andersson JLR, Griffanti L, Douaud G, et al. Image Processing and Quality Control for the first 10,000 Brain Imaging Datasets from UK Biobank. *bioRxiv* [Preprint]. 2017 [cited 2018 Mar 21]. https://doi.org/10.1101/130385.
2. Griffanti L, Zamboni G, Khan A, Li L, Bonifacio G, Sundaresan V,et al. BIANCA (Brain Intensity AbNormality Classification Algorithm): A new tool for automated segmentation of white matter hyperintensities. Neuroimage. 2016;141:191-205. doi:10.1016/j.neuroimage.2016.07.018.
3. Jenkinson M, Beckmann CF, Behrens TEJ, Woolrich MW, Smith SM. FSL. Neuroimage. 2012;62:782-790. doi:10.1016/j.neuroimage.2011.09.015.
4. Mori S, Wakana S, Nagae-Poetscher LM, Van Zijl PCM. MRI Atlas of Human White Matter. Elsevier; 2005
5. Wakana S, Caprihan A, Panzenboeck MM, Fallon JH, Perry M, Gollub RL, et al. Reproducibility of quantitative tractography methods applied to cerebral white matter. Neuroimage. 2007;36:630-644. doi:10.1016/j.neuroimage.2007.02.049.
6. Bycroft C, Freeman C, Petkova D, Band G, Elliott LT, Sharp K, et al. Genome-wide genetic data on ~500,000 UK Biobank participants. *biorXiv* [Preprint]. 2017 [cited 2018 Mar 21]. https://doi.org/10.1101/166298.
7. McCarthy S, Das S, Kretzschmar W, Delaneau O, Wood AR, Teumer A, et al. A reference panel of 64,976 haplotypes for genotype imputation. Nat Genet. 2016;48:1279-1283. doi:10.1038/ng.3643.
8. O'Connell J, Sharp K, Shrine N, Wain L, Hall I, Tobin M, et al. Haplotype estimation for biobank-scale data sets. Nat Genet. 2016;48:817-820. doi:10.1038/ng.3583.
9. Zhan X, Hu Y, Li B, Abecasis GR, Liu DJ. RVTESTS: An efficient and comprehensive tool for rare variant association analysis using sequence data. Bioinformatics. 2016;32:1423-1426. doi:10.1093/bioinformatics/btw079.
10. Woo D, Falcone GJ, Devan WJ, Brown WM, Biffi A, Howard TD, et al. Meta-analysis of genome-wide association studies identifies 1q22 as a susceptibility locus for intracerebral hemorrhage. Am J Hum Genet. 2014;94:511-521. doi:10.1016/j.ajhg.2014.02.012.
11. Willer CJ, Li Y, Abecasis GR. METAL: Fast and efficient meta-analysis of genomewide association scans. Bioinformatics. 2010;26:2190-2191. doi:10.1093/bioinformatics/btq340.
